# Vowel acoustic parameters in speech assessment and rehabilitation of minimally verbal and speech-motor-impaired autistic children: a narrative review

**DOI:** 10.3389/fnhum.2026.1752706

**Published:** 2026-06-19

**Authors:** Yu Chen, Dongfan Chen

**Affiliations:** 1 Xiamen Special Education School, Xiamen, China; 2 Department of Rehabilitation Sciences, Faculty of Education, East China Normal University, Shanghai, China

**Keywords:** articulation disorders, autism spectrum disorder, biofeedback, children, formant analysis, speech rehabilitation, vowel acoustics

## Abstract

Speech production difficulties in autism spectrum disorder (ASD) are heterogeneous and are not uniformly characterized by articulatory impairment across the spectrum. However, some autistic subgroups, particularly minimally verbal children, children with low expressive-language ability, and those with suspected co-occurring speech-motor difficulties, may show atypical vowel-acoustic patterns. Because vowel production can be quantified through measures such as formant frequencies, vowel-space area, duration, and variability, these parameters may offer useful objective information for characterizing speech impairment and informing rehabilitation planning in selected phenotypes. This narrative review critically examined the literature on vowel-acoustic characteristics in autistic children and their possible relevance to rehabilitation-oriented systems. Structured searches of PubMed, Scopus, and Web of Science were conducted for studies published up to September 2025. Evidence suggests that some autistic subgroups may exhibit reduced vowel distinctiveness, greater acoustic variability, and atypical temporal or dynamic speech features, although findings are heterogeneous and do not support a single uniform acoustic profile across ASD. Direct intervention evidence remains limited. Most rehabilitation-related studies are small, exploratory investigations, and the available literature is concentrated largely around Auditory-Motor Mapping Training (AMMT), an indirectly relevant speech-motor intervention that reports vowel-related outcomes but does not directly target vowel-acoustic parameters in isolation.Overall, vowel-acoustic measures are better regarded as candidate quantitative tools for subgroup characterization, monitoring, and rehabilitation planning rather than as universal biomarkers across ASD. Their translational potential is promising but remains insufficiently validated, requiring stronger longitudinal, mechanistic, and intervention research before broad clinical application can be justified.

## 1 Introduction

Autism Spectrum Disorder (ASD) is a complicated neurodevelopmental disorder marked by challenges in social communication, limited interests, and repetitive behaviors. The prevalence of ASD has significantly risen over the past two decades, with current estimates indicating that approximately one in 127 children is diagnosed with ASD globally (Santomauro et al., 2025). Among the various developmental difficulties experienced by some individuals with ASD, speech and language impairments, particularly articulation disorders, remain among the most impactful on daily functioning, hindering intelligibility and reducing communicative efficacy (Rapin and Dunn, 1997; Shriberg et al., 2001). Deficits in articulation not only compromise verbal expression but also intensify social isolation and educational disadvantage, ultimately increasing the functional burden of ASD on children and their families (Tager-Flusberg et al., 2005).

Articulation disorders in some children with ASD often present as inaccurate or inconsistent production of speech sounds, atypical prosody, and reduced precision in motor control of the vocal tract (Vogindroukas et al., 2022). These challenges may manifest as distortions, substitutions, or omissions of phonemes, but in some ASD subgroups, they are frequently accompanied by atypical rhythm, intonation, and reduced speech clarity (Diehl and Paul, 2012). For numerous affected children, traditional speech-language therapy provides gradual enhancements, but fails to fully address the persistent deficits in sound production (Brignell et al., 2018; Simeone et al., 2024). ASD was selected not because articulation disorder is universal or defining within autism, but because speech production profiles in autistic children are highly heterogeneous, and a clinically important subgroup, particularly minimally verbal children and those with co-occurring speech-motor difficulties, may show measurable abnormalities in vowel production. In these subgroups, acoustic vowel parameters may help characterize speech impairment more objectively and may inform intervention design. This review focuses primarily on autistic subgroups in whom vowel-acoustic abnormalities appear most clinically informative, particularly minimally verbal children, low expressive-language groups, and children with marked speech-motor difficulty, rather than assuming a uniform profile across the entire autism spectrum.

Vowels warrant focused examination in this review not because they are more important than consonants or prosody, but because their acoustic properties can be quantified relatively directly through measures such as formant frequencies, vowel-space area (VSA), duration, and token-to-token variability (Kent and Kim, 2003; Kent and Rountrey, 2020). These measures may provide clinically useful information about articulatory-acoustic distinctiveness and may be especially suitable for repeated assessment, acoustic monitoring, and feedback-based intervention (Skodda et al., 2012; Weismer et al., 2001). At the same time, speech intelligibility depends on multiple interacting domains, including consonantal accuracy, prosody, timing, and broader linguistic context. Accordingly, although speech production in autism may be affected at segmental, temporal, and prosodic levels, the present review focuses primarily on segmental vowel-acoustic measures that most directly reflect articulatory-acoustic contrast, including formant structure, vowel-space area, vowel dispersion, and formant variability or trajectories. Temporal and prosodic variables such as duration, rhythm, pitch, and intonation are discussed only insofar as they interact with vowel production or influence the perceptual salience of vowel contrasts, because prosodic abnormality does not by itself necessarily indicate an articulatory disorder (Kent and Rountrey, 2020; Bonneh et al., 2011; Chen et al., 2024; Grossman et al., 2010; Shriberg et al., 2010).

The incorporation of vowel acoustic features into rehabilitation systems has emerged in the last decade, driven by advancements in digital signal processing, machine learning, and human-computer interaction technologies (Brahmi et al., 2024). In contrast to conventional therapeutic approaches that depend significantly on clinician/therapist feedback, acoustic-parameter-based systems can provide prompt, objective feedback to the child. For instance, biofeedback systems that can provide real-time visualizations of vowel formants may enable children to observe their vocal input deviation from the target, facilitating self-correction and motor learning. Mobile applications and computer-based platforms now enable the provision of interventions for neurodevelopmental disorders in clinical settings, schools, and even at home, considering the expanding accessibility of telemedicine (Crişan-Vida et al., 2023). Furthermore, wearable gadgets and AI-powered speech recognition algorithms have been proposed as possible future tools for ongoing monitoring and individualized rehabilitation (Devi et al., 2025).

The significance of this novel method is beyond therapeutic effectiveness. The capacity to accurately quantify vowel characteristics can enhance multidisciplinary research into the speech motor control deficits underlying articulation disorders in ASD (Simeone et al., 2024). By correlating acoustic metrics with neuroimaging, genetic, or neurocognitive data, researchers may achieve novel insights into the mechanisms underlying speech impairment in ASD (Briend et al., 2023; Hiremath et al., 2021). Moreover, acoustic-based rehabilitation systems can play a key role as cost-effective, scalable therapies, particularly beneficial in resource-constrained settings where access to speech-language pathologists is restricted (Davis and Drichta, 1980).

In light of the emerging but still limited literature, this narrative review critically examines the extent to which vowel acoustic parameters currently contribute to understanding speech impairment and informing rehabilitation in selected autistic subgroups, particularly minimally verbal children, low expressive-language groups, and children with marked speech-motor difficulty. Rather than presenting a comprehensive therapy literature, this review synthesizes an early field at the interface of speech acoustics and rehabilitation, with the aim of distinguishing descriptive acoustic findings from clinically actionable targets, clarifying which subgroups are most relevant to the existing evidence, and identifying the empirical and methodological gaps that must be addressed before vowel-based approaches can be translated into robust rehabilitation systems. The review also distinguishes between intelligibility and broader comprehensibility, so that conclusions about vowel-acoustic measures remain appropriately cautious and are not overstated beyond the current evidence base.

## 2 Methods

This narrative review was conducted through structured searches of three databases including PubMed, Web of Science and Scopus for studies published up to September 2025. Search terms included combinations of ‘autism spectrum disorder,’ ‘autistic children,’ ‘vowel acoustics,’ ‘vowel acoustic phonetics’, ‘vowel acoustic metrics’, ‘formant,’ ‘vowel space area,’ ‘speech intelligibility,’ ‘articulation,’ ‘biofeedback,’ and ‘rehabilitation.’ We included peer-reviewed studies addressing vowel-related acoustic characteristics, speech-motor features, and rehabilitation approaches relevant to autistic children with articulation difficulties. We excluded studies unrelated to speech production, non-child populations, and papers lacking relevance to vowel acoustics or intervention implications. The final literature set was selected through iterative discussion among the authors. Because the literature is heterogeneous in design, population, and outcomes, the present article was designed as a narrative critical synthesis rather than a systematic review or meta-analysis.

## 3 Speech and articulation disorders in autism

Speech production in some ASD patients, while heterogenous is marked by a complicated interplay of deficits at the phonetic, phonological, prosodic, and motoric levels. While language impairment itself is not among the criteria for ASD, communication difficulties are common, and articulation disorder is among the most prominent barriers to intelligible speech. Closer examination of the problems of articulation reveals them to extend beyond simple mispronunciations, as they are components of broader deficits of motor planning, sensorimotor integration, and social communication (Schlosser and Wendt, 2008; McCann and Peppé, 2003).

### 3.1 Core characteristics of speech in ASD

Speech errors of some children with autism may be of various types, including distortions, substitutions, omissions, and variable sound output (Vogindroukas et al., 2022; Cleland et al., 2010). For example, they may substitute sounds for each other (“tat” for “cat”), omit sounds or syllables, or distort vowel qualities. However, unlike isolated phonologically disordered children, ASD children are likely to demonstrate abnormal suprasegmental features such as monotonic intonation, atypical rhythm, and improper stress patterns (McCann and Peppé, 2003). The prosodic flaws, therefore, considerably contribute to intelligibility deficits and distinguish the speech of children with autism from that of other clinical groups.

Yet another feature is the variability of speech performance. ASD individuals may successfully reproduce a sound during the first attempt, yet fail to reproduce it systematically in words or tasks (Gomot and Wicker, 2012). The variability complicates testing and treating the child, as the errors cannot be attributed to consistent articulatory patterns but rather reflect underlying motor coordination difficulties.

### 3.2 Motor speech disorders and apraxia in autism

Some autistic children, particularly minimally verbal children or those with suspected co-occurring motor-speech disorders, may exhibit apraxia or dysarthria speech features. However, childhood apraxia of speech (CAS) is not a defining characteristic of ASD and should be distinguished conceptually and clinically from autism itself. In such cases, features such as inconsistent errors, groping, disrupted transitions, vowel distortions, or reduced articulatory precision may reflect co-occurring motor planning or motor execution difficulties rather than autism per se. Comorbidity of autism and CAS has been the topic of vigorous debate despite various studies that estimate that 25–35% of minimally verbal ASD individuals demonstrate apraxia symptoms. Comorbidity draws attention to the need for fine-grained acoustic and motor-based analysis of autistic speech owing to the incompleteness of common phonological theory to explain the disorder’s richness (Cabral and Fernandes, 2021; Chenausky et al., 2023; Guerrera et al., 2025).

Besides apraxia, dysarthria symptoms are also observed in some autistic children, including imprecise consonant pronunciation, reduced vowel distinctness, hyperarticulation of vowels, and typically slurred speech. However, these features should not be generalized across the autism spectrum and may reflect subgroup-specific or comorbid motor speech involvement (Maffei et al., 2024; Belmonte et al., 2013).

### 3.3 Impact on communication and daily functioning

Articulation disorders of ASD go beyond the confines of speech mechanisms. Limited intelligibility impedes the initiation and continuation of social interaction for children, therefore complementing basic autistic symptoms of social isolation and communication impairment (Shumway and Wetherby, 2009). In the educational context, unintelligible speech can reduce classroom participation, obstruct reading and writing development, and reduce educational attainment (Hitchcock et al., 2015). Additionally, behavioral frustration is often concomitant with impairment of articulation, wherein children cannot be understood, sometimes leading to excessive use of nonverbal communication or aggressive behavior (De Giacomo et al., 2016).

The influence of the family is also important. Families of patients with pronounced symptoms of speech disorder experience higher stress levels and poor quality of relationships within the family due to repeated communication breakdowns (Meadan et al., 2010). Rippling effects of such make rehabilitation of speech a clinical imperative of ASD intervention programs.

### 3.4 Co-occurring language and cognitive challenges

In addition, articulation impairment is usually combined with more general language impairment, including delayed learning of basic vocabulary, less syntactic maturity, and pragmatic impairment (Rapin and Dunn, 1997). Cognitive aspects, including reduced working memory and reduced auditory processing, can potentially worsen articulation impairment to the extent that it inhibits the storage and recall of phonological representations by the child (Gonçalves and Monteiro, 2023).

Another complicating factor is heterogeneity. Some of the children with ASD demonstrate relatively normal-sounding speech but less prosody, while others demonstrate severe motor speech impairment with otherwise preserved language abilities. Heterogeneity draws particular attention to the need for individually adapted, parameter-based procedures for treatment and assessment (Demouy et al., 2011).

### 3.5 Clinical assessment of articulation in autism

Acoustic analysis can contribute to speech assessment at multiple levels; however, direct articulatory-acoustic evidence is provided most clearly by segmental vowel measures such as formants, vowel-space area, and related variability indices, whereas prosodic measures provide complementary information about rhythm, stress, and intonation rather than direct evidence of vowel distortion.

Standard test batteries, including standardized tests of articulation, offer limited evidence of the subtle impairment that is typical of ASD. Perceptual clinician assessments, while helpful, are at risk of being inconsistent and subjective. Acoustic analysis measures of vowel formants, durations, and prosodic qualities provide a less subjective and more repeatable system for assessing speech impairment (Asghari et al., 2021). Such objective measures not only detect subtle issues of articulation impairment but also enable accurate change tracking over time during intervention (Wynn et al., 2022).

It is also feasible to utilize recordings of spoken speech to build normative speech databases of people with autism, thus permitting easier cross-comparability of outcomes of various studies and identification of candidate markers of speech impairment in selected autistic subgroups (Briend et al., 2023). Such databases are of significance for creating rehabilitation systems that utilize acoustic feedback.

## 4 Vowel acoustic-parameters in autism research

Research on the acoustic characteristics of vowels in ASD has significantly advanced in the last two decades, yielding essential insights into the articulatory and prosodic attributes of speech in individuals with autism and articulation problems. Previous studies predominantly focused on prosody, intonation, and pragmatic communication elements; however, detailed acoustic analysis has identified atypical vowel-production patterns in some autistic subgroups that may reduce articulatory-acoustic distinctiveness and, in some cases, contribute to reduced speech intelligibility or broader comprehensibility, but the literature is heterogeneous and does not support a single vowel-acoustic profile across the autism spectrum. Reported abnormalities appear to be more pronounced in minimally verbal children and in those with lower expressive-language ability or co-occurring speech-motor difficulty (Simeone et al., 2024; Maffei et al., 2025).

Vowels are central components of spoken language and are particularly suitable for acoustic analysis because their spectral properties can be measured continuously through formant structure. This makes vowel production useful for studying articulatory-acoustic distinctiveness, although speech intelligibility also depends on consonantal accuracy, prosody, timing, and broader linguistic context.

Some studies have reported reduced vowel distinctiveness, atypical formant patterning, or greater acoustic variability in a subgroup of autistic children, although findings vary across subgroups, tasks, and comparison populations. Importantly, not all studies point toward vowel-space reduction; some findings suggest preserved or even expanded articulatory contrasts in certain autistic speakers, indicating that vowel-space configuration should not be interpreted as a uniform ASD feature. The analysis of vowel acoustic characteristics is an effective method for quantifying articulatory precision, identifying reasons for potential impact on intelligibility, and setting measurable treatment objectives (Fogerty and Humes, 2012; Thompson et al., 2023).

This section examines the primary acoustic properties of vowels, their reported changes in ASD patients with articulation problems, their clinical significance, and their importance for developing and evaluating rehabilitation programs for articulation difficulties.

### 4.1 Early observations of speech abnormalities in autism

The initial clinical reports of autistic speech pointed out atypical prosody as “sing-song,” “robotic,” or “flat” speech (McCann and Peppé, 2003). As acoustic phonetic instrumentation was refined further, researchers began to quantitatively assess the spectral and temporal properties of vowels and got empirical support to verify clinical reports of impaired articulation. Shriberg et al. showed that some children with ASD present with both segmental mistakes (misarticulated vowels and consonants) and suprasegmental disturbances (intonation, stress, and rhythm) (Shriberg et al., 2001). These early findings comprised the foundation for subsequent studies that specifically pinpointed vowel production. Formant configuration of vowels, that is, first (F1), second (F2), and third (F3) formants, is reflective of the vocal tract’s resonant quality. F1 is correlated with vowel height (open vs. closed), and F2 is correlated with vowel backness (front vs. back). F3, while less basic to vowel identification, is accountable for fine-grained variations, especially for rounded vowels and rhotic. Formant frequencies are typically measured in Hertz and are the basis of acoustic phonetic analysis (Stevens, 1999). Compressing or reduced distinctness of F1-F2 separation between vowels has been described in more recent ASD research (e.g., reduced vowel space or vowel distinctiveness for minimally verbal or low expressive language ASD children) (Simeone et al., 2024), representing overlapping formant structures that may blur perceptual boundaries. While fewer studies have yet concretely addressed F3 variation for autistic subjects, these findings for F1/F2 strongly support that atypical formant structures represent articulatory imprecision in minimally verbal ASD patients.

### 4.2 Vowel-space area in ASD: heterogeneous evidence and subgroup-specific findings

In addition to vowel space reduction, greater variability in vowel production has been documented in some patients. Children often show less consistency in achieving target articulations, suggesting impairments in motor control and sensorimotor integration. This variability may affect perceptual distinctiveness and contributes to the atypical acoustic profile observed in autistic speech (Simeone et al., 2024; Bishop et al., 2022).

Several studies have reported reduced vowel-space area or reduced vowel distinctiveness in some autistic subgroups, particularly minimally verbal children or those with lower expressive language. However, findings are heterogeneous, and other studies suggest different or less pronounced vowel-space patterns depending on verbal ability, age, language background, task demands, and comparison group. However, these findings should not be generalized across the entire autism spectrum. The literature is heterogeneous, and vowel-space patterns appear to vary depending on verbal ability, developmental level, language background, speech task, and comparison group. Importantly, some autistic speakers may show preserved or even expanded articulatory contrasts, indicating that vowel-space reduction is not a universal feature of ASD. Accordingly, VSA should be interpreted as a context-dependent acoustic measure that may be informative in selected subgroups, rather than as a uniform marker of autistic speech impairment (Fusaroli et al., 2022; Mohanta and Mittal, 2022).

Complementing the measure of VSA, vowel dispersion, which is calculated as the average distance between vowel categories in formant space, provides another lens for assessing acoustic distinctiveness. Low dispersion values indicate centralization of vowels, resulting in speech that sounds less clear and more difficult to understand. Together, reduced VSA and low dispersion capture the characteristic centralization and reduced precision of vowel articulation in some ASD patients, highlighting their value as objective markers of speech deficits (Fletcher et al., 2017).

Reduced vowel-space area (VSA) and lower vowel distinctiveness have been reported in some autistic subgroups, especially in minimally verbal or language-impaired samples, but findings are heterogeneous and should not be generalized across the entire autism spectrum. Vowel-space patterns vary with verbal ability, developmental level, task demands, and comparison group. VSA is typically calculated by plotting the first and second formant frequencies (F1 and F2) of the corner vowels /i/, /a/, and /u/ to form a triangle or quadrilateral in two-dimensional space. A compressed VSA reflects collapsed articulatory distinctions, which in turn may increase the risk of potential impact on intelligibility, especially in more severely affected subgroups (Simeone et al., 2024; Bonneh et al., 2011; Maes et al., 2023).

### 4.3 Vowel formant variability and trajectories

Greater variability in vowel production may reduce the consistency of acoustic cues and thereby affect speech clarity or, in some cases, intelligibility, especially in more severely affected subgroups. Chenausky et al. discovered that autistic children, particularly those with co-occurring apraxia of speech, produced inconsistent F1 and F2 values after repeated tries at the same vowel (Chenausky et al., 2016). Such inconsistency affects intelligibility because listeners rely on consistent acoustic cues to appropriately recognize phonemes.

In addition to static vowel measurements, analysis of formant trajectories (the dynamic transitions between consonants and vowels) has shown further irregularities. Whereas typically developing children have smooth and clearly sloped trajectories that suggest fine articulatory coordination, children with ASD have flatter, less regular, or less smooth trajectories. These discrepancies point to poor coarticulatory control and difficulty with fine-grained motor planning. Variability in static formant frequencies and anomalies in formant trajectories support the concept that difficulties in motor control and sensorimotor integration may contribute to articulation problems in autism subgroups (Talkar et al., 2020; Simarro Gonzalez et al., 2024).

It should be noted that, comparisons across studies should be interpreted cautiously, because reported formant values and derived measures such as VSA depend not only on participant performance but also on extraction procedures, including software or scripts used, parameter settings, normalization choices, and whether measurements are taken at static points or across dynamic trajectories. Studies using midpoint or other static measurements are not directly equivalent to studies examining formant trajectories or dynamic spectral change, and these differences may partly account for inconsistent results in the literature. Accordingly, any clinical interpretation of vowel-acoustic measures must take into account not only phenotypic heterogeneity within autism but also methodological heterogeneity across studies.

### 4.4 Temporal parameters: vowel duration and speech rhythm

Temporal and prosodic measures should be interpreted separately from direct segmental vowel-articulatory measures. Vowel duration may relate to temporal control of speech production and can affect the realization of vowels, whereas rhythm, pitch, stress, and intonation belong primarily to the suprasegmental organization of speech. These domains may interact perceptually with vowel clarity and intelligibility, but altered prosody does not necessarily imply an underlying articulatory disorder (Vogindroukas et al., 2022; Patel et al., 2020).

Vowel duration has been a frequent target of ASD speech research, as temporal control is critical for speech rhythm and fluency. Grossman et al. found that some autistic children frequently produce vowels that are either lengthy or inconsistent in length, disrupting the natural cadence of speech. Prolonged durations may represent compensatory attempts for articulatory instability, whereas variability indicates temporal regulation issues (Grossman et al., 2010). Chenausky et al. have noticed that such irregularities are more visible in minimally verbal children with autism who also exhibit apraxia characteristics, emphasizing the link between motor planning deficiencies and temporal dysregulation. These data support vowel duration as both a diagnostic marker and a therapeutic target for management (Chenausky et al., 2016).

Beyond static formant measurements, temporal and prosodic characteristics reveal further details regarding vowel production. Duration, whether at the vowel or syllable level, is essential for conveying stress, rhythm, and, in some languages, phonemic distinction. Longer or more varied vowel durations in patients, particularly in structured tasks or prosody imitation, have been associated with decreased fluency and less natural-sounding speech (Diehl and Paul, 2012; Paul et al., 2008).

Abnormalities are not limited to time; abnormal pitch patterns, such as altered mean levels, decreased variability, and restricted range, have been thoroughly reported in both prosodic and emotional contexts. Although the exact relationship between pitch variation and vowel clarity is unclear, these aberrations contribute to autism’s prosodic profile in patients with articulation difficulties. Furthermore, children with ASD with speech problems demonstrate reduced or inconsistent intensity modulation, resulting in weaker contrasts in stress and prominence and a perceptual perception of flatness or monotonicity (Bonneh et al., 2011; Asghari et al., 2021).

Disruptions in duration, pitch, and intensity emphasize the importance of temporal and prosodic aspects in determining vowel clarity and overall intelligibility, emphasizing their diagnostic and therapeutic use (Grossman et al., 2010).

### 4.5 Interaction with prosody and intonation

Although vowels are primarily defined by spectral properties, they are deeply intertwined with prosody and intonation. For instance, Diehl and Paul found that some children with ASD have longer utterances and greater pitch variance and range even when using correct prosody tasks, indicating that prosodic abnormalities are present in fine acoustic detail (Diehl and Paul, 2012). Meta-analytic evidence further shows consistent differences in pitch mean, pitch range, pitch variability, and duration between ASD and typically developing speakers. These prosodic differences may interact with vowel articulation: abnormal stress, unusual intonation, or irregular duration may reduce the perceptual salience of vowel contrasts, making speech less intelligible. This complexity means that isolating vowel deficits in these patients requires examining them in the broader prosodic context, rather than as purely segmental units (Asghari et al., 2021). However, prosodic abnormalities such as atypical rhythm, stress, pitch, or intonation may affect speech naturalness and intelligibility, but they should not be treated as equivalent to direct articulatory-acoustic evidence of vowel distortion.

### 4.6 Cross-linguistic and developmental findings

Research across languages suggests that some trends in vowel/acoustic anomalies emerge in ASD speech (e.g., reduced formant-based vowel distinctiveness, hyperarticulation of vowels, greater token-to-token variability, unusual duration), though results vary by language, age, sex, and severity. Therefore, these characteristics should be treated as potential moderators when interpreting vowel-acoustic findings. For example, in a study of US English, children with ASD with lower expressive language showed significantly less acoustic distance between vowels, which predicted expressive ability (Simeone et al., 2024). Meta-analytic and cross-linguistic work (notably English and Danish) supports that acoustic/prosodic abnormalities are moderately reliable markers, though no consensus “vowel-anomaly profile” applies to all individuals (Fusaroli et al., 2022). Early measures of vocal output and acoustic quality in some toddlers with ASD are correlated with subsequent language development, suggesting vowel/acoustic parameters may have predictive value, but evidence is still scarce, especially across more diverse linguistic contexts (e.g., Mandarin, Hebrew) and with larger, longitudinal cohorts (McDaniel et al., 2020). Moreover, other participant characteristics such as age, verbal level, sex distribution, and language background should be treated as potential moderators when interpreting vowel-acoustic findings.

### 4.7 Candidate quantitative markers and clinical implications

Several studies suggest that vowel and speech anomalies tend to be more pronounced in autistic children who have severe language impairment or who are minimally verbal. For example, Chenausky et al. found that minimally verbal children with ASD exhibited especially high variability (within and between tokens) and reduced vowel space area (Chenausky et al., 2021). Maffei et al. similarly document greater deficits in speech precision and consistency in low-verbal/minimally verbal ASD groups (Maffei et al., 2024). The evidence for particularly severe vowel distortions in children with ASD plus apraxia is less clear, as Shriberg et al. did not find strong statistical support for CAS in their sample (Shriberg et al., 2011). These findings highlight the heterogeneity in ASD speech and underscore the need for individual assessments of acoustic measures.

Vowel-acoustic measures may serve as candidate quantitative markers for subgroup characterization, monitoring, and rehabilitation planning, particularly in minimally verbal or severely language-impaired autistic children, rather than as universal markers across ASD. Some multivariate studies suggest that combinations of acoustic features may help distinguish certain autistic speech samples from typically developing comparison groups, but these findings are heterogeneous and do not establish stable, vowel-specific diagnostic biomarkers across the autism spectrum (Briend et al., 2023; Fusaroli et al., 2022; Mohanta and Mittal, 2022). These biomarkers hold potential for early detection of speech deficits, tracking therapeutic progress, and stratifying individuals for targeted interventions. However, further large-scale, longitudinal, multilingual studies are required to validate how stable these vowel−/formant-based measures are over time, and how well they predict developmental speech/language trajectories. However, it should be noted that the most direct candidate quantitative markers discussed in this review are segmental vowel-acoustic measures linked to articulatory contrast; prosodic measures are considered complementary contextual features rather than primary articulatory biomarkers.

All in all, findings from vowel acoustic research have several implications for clinical practice. Incorporating vowel analysis into diagnostic protocols can provide objective measures of articulatory precision, supplementing clinician ratings. Moreover, vowel clarity and distinctiveness can serve as explicit goals in speech-language therapy. Thus, real-time visualization of vowel formants offers a promising method for engaging children in individualized therapy and promoting self-monitoring. Because speech production in autism is heterogeneous, vowel-acoustic measures are best understood as subgroup-sensitive quantitative tools for characterization and monitoring, rather than universal diagnostic biomarkers.

## 5 Auditory-motor mapping training and vowel-related speech outcomes

The intervention literature directly relevant to this review remains limited and is currently concentrated in a small number of AMMT studies. Accordingly, this section should not be interpreted as a comprehensive review of multiple vowel-based rehabilitation systems, but rather as a preliminary discussion of one indirectly relevant intervention approach with vowel-related outcomes. The currently available intervention literature does not yet establish a broad field of vowel-specific rehabilitation systems in autism; rather, it offers early translational evidence from a small number of pilot or proof-of concept speech-motor intervention studies that include vowel-related outcomes.

Auditory-Motor Mapping Training (AMMT) was included in this review not because it directly trains vowel formants or vowel-space targets in isolation, but because it is one of the few speech interventions studied in minimally verbal autistic children that reports vowel-related speech outcomes alongside broader syllable and consonant measures. Its primary rationale is speech-motor and auditory-motor integration, supported by intonation and rhythmic cueing, rather than vowel acoustics per se. Accordingly, AMMT is discussed here as an indirectly relevant intervention whose results may have implications for vocalic intelligibility, but not as a pure vowel-acoustic rehabilitation system.

AMMT was originally developed to facilitate speech output through paired auditory and motor cues, using intoned bisyllabic stimuli, rhythmic structure, and bimanual movements to support speech production. Its putative mechanism relates more directly to auditory-motor integration and speech-motor planning than to isolated vowel training. Therefore, its relevance to the present review lies in whether such intervention produces measurable improvements in vowel-related speech output, not in direct manipulation of vowel-acoustic parameters such as formant targets (Chenausky et al., 2017; Wan et al., 2011).

To our knowledge, three studies examining the effectiveness of AMMT in children with ASD and speech disorders were published, including designs from proof-of-concept to controlled comparisons. Taken together, these studies show that AMMT might improve speech production in autistic children across different levels of verbal ability, from those who are minimally verbal to more verbal but still delayed children, and that these possible clinically relevant gains are greater than those achieved with comparable therapies that use spoken repetition alone, without intonation or rhythmic support. The study characteristics and main findings were summarized in Table 1.

**Table 1:** Rehabilitation systems based on vowel acoustic parameters for autistic children.

Study, year	Design	Sample size, population	Age	Intervention	Comparator	Acoustic-related outcomes	Main findings
Wan et al. (2011)	Proof-of-concept, single-subject design	*N* = 6 non/minimally verbal children with ASD	5–9 y	AMMT (intoned bisyllables + bimanual drumming), 40 sessions (5×/week × 8 weeks)	None (single-arm)	% Vowels Correct (plus % syllables, consonants)	Significant gains from baseline on speech production indices including vowel accuracy after 40 sessions.
Chenausky et al. (2016)	Controlled trial with matched pairs	*N* = 23 minimally verbal children with ASD (7 AMMT vs. 7 SRT)	3–9 y	AMMT ≥25 sessions (5×/week, 45 min)	SRT (non-intoned repetition), ≥25 sessions	% Vowels Correct, % Consonants Correct, % Syllables Approximated	At 25 sessions, AMMT > SRT; vowel accuracy improved about 17–19% from baseline in AMMT groups; pre-treatment phoneme imitation predicted response.
Chenausky et al. (2017)	Case comparison of minimally verbal and more-verbal pairs	4 male children with ASD (2 minimally verbal vs. 2 more verbal children)	4–6	AMMT, 25 sessions (5×/week, 45 min)	SRT	Per-session speech accuracy including vowels	Demonstrated applicability to more-verbal ASD with improvements and hypothesized neural mechanisms

ASD = autism spectrum disorder; SRT: Speech repetition therapy; AAMT: Auditory-motor mapping training.

The preliminary proof-of-concept research conducted by Wan et al. assessed six nonverbal children aged 5 to 9 years who had undergone a minimum of 18 months of traditional speech treatment without attaining intelligible speech. Thus, they were included and completed 40 intensive sessions of AMMT over a duration of 8 weeks. Post-treatment, all children exhibited substantial advancements in the production of consonant-vowel pairings, with enhancements extending to untrained words and phrases. Significantly, these improvements were maintained at follow-up, indicating both the efficacy of the intervention and its potential to stimulate initial speech in children who had previously been completely nonverbal (Wan et al., 2011).

Chenausky et al. conducted a controlled trial comparing AMMT with Speech Repetition Therapy (SRT), a non-intoned control condition. Twenty-three minimally verbal children with ASD (mean age 6 years and 5 months) underwent a minimum of 25 therapy sessions. Seven subjects undergoing AMMT were paired based on age and verbal proficiency with seven children getting SRT. Within the entire AMMT group, children exhibited significant improvements following 25 sessions, with mean enhancements of 19.4% in approximated syllables, 13.8% in accurate consonants, and 19.1% in correct vowels compared to baseline measurements. In the matched subgroup, AMMT individuals significantly surpassed their SRT counterparts, achieving a 29.0% enhancement in syllables approximated, in contrast to 3.6% in the SRT group, with similarly pronounced disparities in consonant and vowel correctness. Statistical analysis demonstrated that a considerably greater number of AMMT participants attained substantial improvement, with pre-treatment phoneme imitation ability identified as a predictor of treatment responsiveness. The data indicate that AMMT yields clinically meaningful enhancements in speech production, surpassing the outcomes of traditional repetition-based therapy (Chenausky et al., 2016).

A second study broadened this comparison to encompass more verbal children. Four male volunteers, aged 4 to 6 years, were matched based on verbal proficiency and allocated to either AMMT or SRT for 25 sessions. In the minimally verbal pair, AMMT demonstrated superior enhancements in syllables and consonants correct compared to SRT, aligning with previous research findings. Nonetheless, the most pronounced impacts were noted in the more verbal pair. The children undergoing AMMT had substantial improvements in syllables accurate per stimulus (Cohen’s d > 1.9), whereas the SRT participant experienced a minor decrease during the same timeframe. Improvements extended to untrained stimuli, underscoring the extensive influence of AMMT on speech development. The effect sizes for the more verbal children were much bigger than controls, indicating that baseline verbal ability may influence treatment responsiveness (Chenausky et al., 2017).

Because the available AMMT studies report combined gains across vowels, consonants, and syllable production, the current evidence is more consistent with broader speech-motor improvement than with isolated remediation of vowel-acoustic structure. Taken together, the currently available AMMT studies suggest possible gains in speech production, including vowel-related outcomes, particularly in minimally verbal ASD and small exploratory subgroup comparisons. Although some preliminary findings suggest possible applicability beyond minimally verbal samples, the current evidence is insufficient to generalize across verbal profiles. Totally, the evidence base remains limited to a small number of studies with small samples, restricted controls, and sparse long-term follow-up. Therefore, AMMT should be interpreted as preliminary speech-motor intervention evidence with vowel-related relevance, rather than as definitive support for broad rehabilitation claims across autistic children with speech disorder. When vowel-acoustic abnormalities are associated with suspected co-occurring motor-speech disorders, rehabilitation should be interpreted as targeting the child’s specific speech-motor profile rather than autism in a general or syndrome-level sense.

The rationale for technology-assisted vowel intervention is not that vowels are uniquely central to autism, but that vowel-acoustic parameters can provide measurable external targets for auditory-motor practice in some children with speech production difficulty. This mechanism is likely to be most relevant in selected phenotypes, particularly minimally verbal children or those with co-occurring speech-motor impairment, and remains a translational hypothesis that requires stronger empirical validation.

## 6 Challenges and limitations

**Figure 1:**
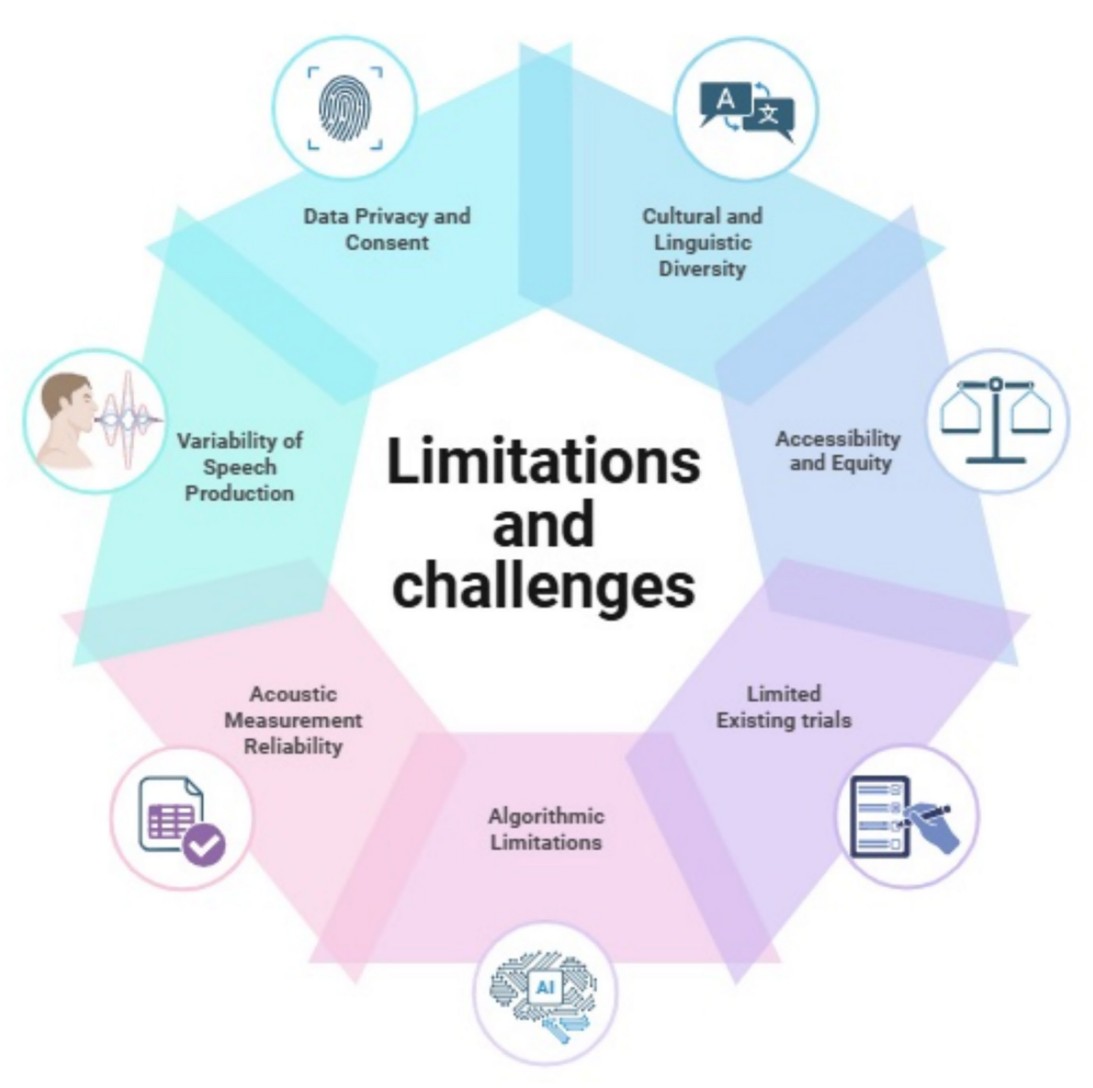
Limitations and challenges for rehabilitation based on vowel acoustic parameters for autistic children.

Despite the potential of vowel acoustic-parameter-based rehabilitation systems to enhance speech intelligibility in autistic children with articulation difficulties, various technological, clinical, and practical obstacles presently hinder their broad implementation (Figure 1). Comprehending these obstacles is essential for enhancing intervention options and directing future research.

Children’s speech has inherent variability owing to developmental alterations in vocal tract anatomy and motor control (Kent and Rountrey, 2020; Murdoch et al., 2012). This variability is heightened in autism, characterized by uneven sound output and variable prosody (Broome et al., 2023). Thus, vowel formants and VSA may vary not just among children but even within the same child throughout different sessions (Heller Murray et al., 2022). This intra-speaker heterogeneity affects the development of effective rehabilitation systems and hinders the establishment of normative baselines. Furthermore, acoustic studies are influenced by environmental variables, including microphone type, recording distance, and ambient noise. Even minor adjustments in recording configurations can modify the recorded formant values. Although laboratory-grade equipment guarantees accuracy, in clinical and domestic settings, there are concerns over measurement consistency. In the absence of established protocols, results may pose challenges for comparison among studies or for use in clinical practice. Furthermore, machine learning and ASR systems developed on standard adult speech frequently exhibit suboptimal performance when utilized for children or individuals with speech impairments (Broome et al., 2023). Autistic speech, characterized by unusual prosody and articulatory patterns, presents further difficulties for precise vowel recognition. Currently available algorithms may misclassify vowel tokens, constraining their effectiveness in real-time feedback scenarios.

On the other hand, the majority of interventional studies on vowel acoustic-based rehabilitation in autism are limited to pilot trials with small sample sizes. Variability in participant age, autism severity, and concurrent speech impairments further constrains generalizability. Thus, the absence of extensive, multicenter trials hinders the ability to reach definitive findings regarding efficacy. Speech-language pathologists often depend on conventional perceptual techniques, and numerous practitioners lack proficiency in acoustic analysis. Incorporating vowel acoustic-parameter systems into standard therapy necessitates not just novel instruments but also further professional advancement. Furthermore, therapies should be aligned with comprehensive therapeutic objectives, such as pragmatics and language comprehension, rather than exclusively concentrating on vowel accuracy. Importantly, children with autism vary widely in cognitive, motor, and sensory profiles. For minimally verbal children or those with co-occurring intellectual disability, the cognitive demands of interpreting visual biofeedback or sustaining attention in feedback tasks may be challenging (Guerrera et al., 2025). Sensory sensitivities (to sound, tactile stimulation, screens, headphones, microphones) are common in ASD and may reduce comfort or willingness to use such equipment, which in turn can affect therapy compliance (Pfeiffer et al., 2019). These factors should be considered when designing acoustic/feedback-based interventions.

In addition, many of the current vowel-based rehabilitation systems require specialized equipment and software not readily available in community clinics or schools. Commercially available biofeedback equipment can be expensive, thus restricting access for families from lower socioeconomic backgrounds. Maintaining affordability and scalability presents a significant problem. Languages exhibit significant variation in their vowel inventory. For instance, English possesses a more extensive range of vowel distinctions, whereas Spanish and Japanese exhibit fewer distinctions (Bradlow, 1995; Yazawa et al., 2023). Rehabilitation methods designed for English may not be applicable to other languages, necessitating adjustments to formant objectives to accommodate dialectal and cross-linguistic variations. In the absence of culturally appropriate techniques, numerous children face the risk of exclusion from the advantages of vowel acoustic-based therapies. Last but not least, systems that collect and store recordings of children’s voices pose privacy issues. Protecting this sensitive data necessitates rigorous compliance with ethical standards and parental consent protocols. Furthermore, families must be thoroughly apprised about the methods of storage, analysis, and potential dissemination of recordings in research or clinical settings.

These limitations highlight the necessity for interdisciplinary cooperation among engineers, doctors, linguists, and ethicists to create standardized, accessible, and equitable interventions. Despite these obstacles, the field continues to evolve rapidly. Progress in resilient signal processing, pediatric-specific automatic speech recognition, and affordable mobile health technology provides intriguing avenues to address numerous existing limitations. Confronting these issues is crucial to convert vowel acoustic analysis from research into a therapeutically feasible, scalable method for enhancing speech outcomes in children with autism and speech disorder.

## 7 Future directions

Future technology-enhanced approaches may help refine individualized assessment and intervention, but their clinical value should not be assumed in advance of stronger empirical validation. Biofeedback systems, machine-learning-assisted analysis, mobile platforms, and gamified environments are best regarded as promising implementation pathways whose usefulness will depend on phenotype-specific applicability, measurement reliability, and demonstration of meaningful long-term speech and communication outcomes.

The expanding body of evidence on vowel acoustic-parameter-based rehabilitation systems underscores both the potential and the constraints of existing methodologies. Future advancements in this field will rely on the utilization of technology innovations, therapeutic breakthroughs, and interdisciplinary collaborations. Numerous prospective avenues are particularly vital for enhancing the effectiveness, scalability, and equity of vowel acoustic-based therapies for autistic children with articulation problems. A highly promising avenue is the advancement of individualized therapeutic systems. Children with autism exhibit markedly diverse profiles of speech and language deficits. Therefore, a one-size-fits-all rehabilitation strategy is unlikely to be effective. Future systems ought to customize therapeutic objectives according to the child’s unique acoustic profile, including particular formant distortions, diminished vowel space, or irregular vowel durations. Machine learning can serve a pivotal function, facilitating adaptive algorithms that modify targets during child development. For instance, biofeedback systems could gradually shift from broad vowel contrasts to fine-grained distinctions as a child’s accuracy improves. This personalized calibration could optimize therapeutic benefits while maintaining children’s motivation.

Recent advancements in digital health and AI may support future implementation of distinctive prospects to improve vowel-centric therapies. Novel neural network models trained on extensive datasets of child speech, including atypical features, might enhance vowel identification accuracy. This will enable real-time feedback systems to function reliably in clinical and domestic settings. Immersive environments can convert monotonous articulation exercises into engaging, gamified experiences. For example, vowel precision may activate functions in a virtual game, rendering therapy both engaging and efficient. Smartphones and tablets might provide affordable interventions beyond clinical settings, enhancing accessibility for families in marginalized areas. Integrating vowel-tracking algorithms into applications would enable children to practice at home, with their progress automatically recorded for therapists, if validated. Wearable technology, such as portable microphones or head-mounted sensors, might continuously capture speech samples in natural settings, facilitating ecological evaluations of articulation outside the clinic. These technologies remain promising but insufficiently tested in the clinical settings.

Although several technologies appear promising, their clinical implementation will require more than technical innovation alone. Future work should prioritize standardized protocols for acoustic recording, formant extraction, normalization, and reporting so that findings can be compared across studies and translated more reliably into practice. In parallel, speech-language pathologists will need accessible tools and targeted training to incorporate acoustic analysis into routine care. User-friendly interfaces that automate acoustic computation while providing clinically interpretable feedback may facilitate adoption in both research and community settings. These implementation efforts should accompany, not replace, stronger efficacy data from larger and better-controlled studies.

The majority of current studies are limited in scope and exploratory in nature. Extensive, multicenter randomized controlled trials are essential to provide substantial evidence for vowel-based therapies. Such trials should assess not only speech outcomes but also functional communication, social engagement, and quality of life. Longitudinal designs will be crucial for evaluating whether early intervention in vowel clarity forecasts improved developmental outcomes. Future systems must also tackle equity concerns. Interventions must be economical, expandable, and culturally appropriate. Developing therapies compatible with low-cost equipment will enhance accessibility beyond specialized clinics. Vowel inventories vary significantly among languages, necessitating tailored rehabilitation approaches. The establishment of language-specific vowel goals will be crucial for global application. Systems must consider sensory sensitivities, motor limitations, and cognitive diversity prevalent in autism, enabling successful participation for children throughout the spectrum. The progress of vowel acoustic-parameter rehabilitation depends on the collaboration of engineers, clinicians, linguists, and neuroscientists. This interdisciplinary approach will improve interventions and deepen our understanding of speech development and rehabilitation in autism.

## 8 Conclusion

In conclusion, the present review should be interpreted as a critical synthesis of an emerging and still insufficiently developed area rather than as evidence that vowel-acoustic rehabilitation systems for autism are already well established. Available studies suggest that some autistic subgroups, particularly minimally verbal children and those with more severe language or speech-motor impairment, may show reduced vowel distinctiveness, increased variability, and atypical temporal or dynamic speech features; however, the literature remains heterogeneous and does not support a single uniform acoustic profile across the autism spectrum. Vowel-acoustic measures may therefore be most useful as subgroup-sensitive quantitative tools for characterization, monitoring, and rehabilitation planning rather than as universal diagnostic biomarkers. Although technology-assisted analysis and feedback systems, including biofeedback, mobile platforms, and AI-assisted tools, may have translational potential, direct intervention evidence in autistic children remains limited and is concentrated mainly in a small number of AMMT-related studies. Important questions regarding mechanism, durability, phenotype specificity, accessibility, and cross-linguistic generalizability remain unresolved, and future progress will depend on standardized methods, affordable and culturally adaptable tools, and larger multicenter longitudinal and interventional studies.
